# A probabilistic atlas of finger dominance in the primary somatosensory cortex

**DOI:** 10.1016/j.neuroimage.2020.116880

**Published:** 2020-08-15

**Authors:** George C. O’Neill, Ayan Sengupta, Michael Asghar, Eleanor L. Barratt, Julien Besle, Denis Schluppeck, Susan T. Francis, Rosa M. Sanchez Panchuelo

**Affiliations:** a Sir Peter Mansfield Imaging Centre, School of Physics and Astronomy, University of Nottingham, Nottingham, UK; b Wellcome Centre for Human Neuroimaging, Institute of Neurology, University College London, London, UK; c Wolfson Brain Imaging Centre, Department of Clinical Neurosciences, University of Cambridge, Cambridge, UK; d Department of Psychology, Royal Holloway, University of London, Egham, UK; e Department of Psychology, American University of Beirut, Beirut, Lebanon; f School of Psychology, University of Nottingham, Nottingham, UK; g NIHR Nottingham Biomedical Research Centre, University of Nottingham, Nottingham, UK

**Keywords:** Atlas, Somatosensory cortex, Digits, fMRI, Ultra-high field, Open science

## Abstract

With the advent of ultra-high field (7T), high spatial resolution functional MRI (fMRI) has allowed the differentiation of the cortical representations of each of the digits at an individual-subject level in human primary somatosensory cortex (S1). Here we generate a probabilistic atlas of the contralateral SI representations of the digits of both the left and right hand in a group of 22 right-handed individuals. The atlas is generated in both volume and surface standardised spaces from somatotopic maps obtained by delivering vibrotactile stimulation to each distal phalangeal digit using a travelling wave paradigm.

Metrics quantify the likelihood of a given position being assigned to a digit (full probability map) and the most probable digit for a given spatial location (maximum probability map). The atlas is validated using a leave-one-out cross validation procedure. Anatomical variance across the somatotopic map is also assessed to investigate whether the functional variability across subjects is coupled to structural differences. This probabilistic atlas quantifies the variability in digit representations in healthy subjects, finding some quantifiable separability between digits 2, 3 and 4, a complex overlapping relationship between digits 1 and 2, and little agreement of digit 5 across subjects. The atlas and constituent subject maps are available online for use as a reference in future neuroimaging studies.

## Highlights

• Here we present a probabilistic atlas of the hand and digits in the primary somatosensory cortex.
• The atlas was created by combining multiple 7 T fMRI datasets where a travellingwave paradigm to map the digits was employed.
• Maps are reproducible across sessions and show large overlap across subjects in template anatomical spaces
• Maps are reproducible across sessions and show large overlap across subjects inThe atlas shows that non-invasive electrophysiological data can be separated according to digits stimulated.
• The atlas is publically available and will be refined with additional data over time.The atlas is publically available and will be refined with additional data over time.

## 1 Introduction

Functional magnetic resonance imaging (fMRI) has proved to be a valuable tool for the non-invasive in-vivo study of orderly topographic organization of different cortical areas in humans. It has revealed the retinotopic organization of the visual cortex (DeYoe et al., 1996; Engel, 1997; Sereno et al., 1995; Wandell et al., 2007), tonotopic organization in the auditory cortex (Da Costa et al., 2011; Formisano et al., 2003; Moerel et al., 2018, 2012; Saenz and Langers, 2014) and the cortical representation of body parts (Akselrod et al., 2017; Sanchez Panchuelo et al., 2018), particularly the digits of the hand (Besle et al., 2013; Sanchez Panchuelo et al., 2010; Schweisfurth et al., 2015, 2014; Stringer et al., 2011; van der Zwaag et al., 2015) in the primary somatosensory cortex (S1). Due to the fine architecture of the cortical representation of the digits of the hand in the post-central gyrus (Geyer et al., 2000), somatotopic mapping is more challenging than retinotopic and tonotopic mapping, in terms of both the spatial resolution of cortical maps and the statistical power (Francis et al., 2000; Gelnar et al., 1998; Huang and Sereno, 2007; Kurth et al., 2000; Nelson and Chen, 2008; Overduin and Servos, 2004; Weibull et al., 2008). With the advent of ultra-high-field (UHF) MR scanners, operating at 7 ​T and above, high spatial resolution fMRI has provided robust maps of the representation of all the digits of the hand in primary somatosensory cortex in individual subjects (Besle et al., 2014, 2013; Martuzzi et al., 2014; Sanchez Panchuelo et al., 2010).

Inspired by recent work to generate probabilistic maps of visual topographic areas (Wang et al., 2015), this study aims to generate a probabilistic atlas of individual digit representations in the primary somatosensory cortex in both standard volume space (MNI-152) and standard surface space (MNI-305). These probabilistic maps provide a method to define the likelihood of a given coordinate being associated with a particular functionally defined digit over a population of subjects. These maps can then be used to infer the localisation of the digits in the primary somatosensory cortex of any independent data set. Probabilistic maps provide a particular advantage in the somatosensory domain, as defining somatotopic maps in individual subjects requires additional MR-compatible stimulation equipment and data must be acquired at high or ultra-high field for sufficient spatial resolution to define the individual digits.

Here, we present probabilistic maps of each of the five digits in contralateral S1 from 7 ​T travelling wave fMRI data collected in 22 right handed subjects in response to vibrotactile stimulation of the tips of both hands (Besle et al., 2013; Sanchez Panchuelo et al., 2010). Binary maps of each digit of the hand are generated, individually for each participant and hand, and transformed into standardised volume space (Collins et al., 1994) and surface space (Fischl et al., 1999b) to generate a probabilistic atlas of digit representations in contralateral S1. We then assess a number of metrics associated with these maps in both volume and surface space to determine the likelihood of a given spatial position being assigned to a digit (full probability map), the most probable digit for a given spatial location (maximum probability map), and used a leave-one-out cross-validation procedure to test the generalisability of the atlases. Using these metrics, we investigate variations in the spatial localisation and size of the individual digit atlases, and we address which of the digits can be better co-localized using the atlases. These probabilistic atlases are made freely available in formats compatible with major fMRI analysis packages.

Functional maps are linked to the underlying structure. The primary somatosensory cortex is subdivided structurally into four Brodmann areas (3a, 3b, 1 and 2), classified based on their distinct cytoarchitectonic profiles (Brodmann, 1909), with each of these areas containing a somatotopic map of the contralateral body side (Kaas et al., 1979). Since ultra-high resolution MRI provides the ability to resolve intracortical contrast in-vivo (e.g. area specific signatures have been revealed in areas 3b,1, 2 and 4 using quantitative T1; Dinse et al., 2015), there has been an increasing interest into linking functional cortical fields with distinct structural characteristics. We have shown that anatomical features (cortical thickness and myelin sensitive measures) vary across functionally defined parcellations of the primary somatosensory cortex (Sánchez-Panchuelo et al., 2014). Structural variations defined either by myelination (Glasser et al., 2016; Kuehn et al., 2017) or the grosser morphometry of the folding patterns (Germann et al., 2019) have also been observed within Brodmann areas, with these structural subdivisions correlating with distinct functional cortical fields defined by the topography to specific body sites. Given the link of each digit representation with the structural features of the S1, and given that registration across subjects is performed based on anatomical landmarks, it is possible that inter-subject anatomical variability may play a role in driving the probabilistic digit maps. To investigate this, we compute the degree of anatomical variance across the somatotopic maps.

## 2 Materials and methods

Functional MRI data were pooled from four studies (Barratt, 2018; Granga Espiritu Santo, 2018; Sanchez Panchuelo et al., 2016, Sanchez Panchuelo et al., 2018) collected between 2015 and 2018 on the same 7 ​T Achieva MR system (Philips Healthcare; Best, Netherlands) using a head volume transmit coil and a 32-channel receive coil (Nova Medical: Wilmington, MA). Experimental procedures for all studies were approved by the University of Nottingham Medical School’s Ethics Committee. All subjects gave written informed consent and subjects had no history of neurological disorders.

To generate the digit probabilistic atlas, only those subjects who had completed somatotopic mapping of both the left and right hand were included. This resulted in the inclusion of data from 22 right handed healthy human subjects (equal biological sex distribution, age 29 ​± ​9 years). In order to assess reproducibility of the somatotopic maps, four of these twenty-two subjects subsequently participated in an additional scan session to generate a second digit somatotopic map for both the left and right hand.

### 2.1 Paradigm and acquisition

Vibrotactile stimulation was delivered to a ~1 ​mm^2^ area of the skin of the distal phalanges (fingertips) of the left or right hands using five independently controlled piezo-electric devices (Dancer Design, St. Helens, UK). A ‘travelling wave’ paradigm was used to sequentially stimulate each of the five digits of the left or right hand, in either a forward (from digit 1 to digit 5) or backward (from digit 5 to digit 1) ordering. Each vibrotactile stimulation lasted 4 ​s and consisted of bursts of 0.4 ​s duration at 30 ​Hz stimulation frequency separated by 0.1 ​s gaps so as to limit habituation effects (Tommerdahl et al., 2005, 1999). A stimulation cycle across the five digits lasted 20 ​s. Functional scans consisted of 8–12 cycles and were repeated twice for each hand, alternating between forward and backward ordering.

Functional MRI data were acquired using T2∗-weighted, multi-slice, single-shot gradient echo–echo planar imaging (GE-EPI) at either 1.25 ​mm (n ​= ​10) or 1.5 ​mm (n ​= ​12) isotropic spatial resolution. 26 slices were acquired for the 1.5 ​mm isotropic resolution data. The 1.25 ​mm isotropic resolution data was collected using a Simultaneous Multi-Slice (SMS) factor of 2 to acquire 52 slices covering SI and SII. All other imaging parameters were identical: repetition time (TR) 2 ​s, echo time (TE) 25 ​ms, flip angle (FA) 75°, field of view of 192 ​× ​192 ​mm^2^ in the anterior-posterior and right-left directions, SENSE acceleration factor 3 in the anterior-posterior direction. Functional runs were followed by the acquisition of a high-resolution, T2∗-weighted axial FLASH image with the same slice prescription and coverage as the functional data (0.5 ​× ​0.5 ​mm^2^ in-plane resolution; TE/TR ​= ​9.3/458 ​ms, FA ​= ​32°, SENSE factor ​= ​2), acquired to allow subsequent registration to a structural whole head 1 ​mm isotropic resolution T1-weighted reference volume. For each participant, the structural T1-weighted anatomical image had been previously acquired using either a phase sensitive inversion recovery sequence (PSIR; Hou et al., 2005; Mougin et al., 2016; Van de Moortele et al., 2009; with linear phase encoding order, TE/TR 3.7/15 ​ms, FA 8°, inversion times 778 and 2500 ​ms, using a tailored RF TR-FOCI inversion pulse; Hurley et al., 2009) or MPRAGE scan (linear phase encoding order, TI ​= ​996 ​ms, TE/TR 3.4/7.4 ​ms, FA 8°).

In addition to participating in MR scanning sessions, all subjects completed the Edinburgh Handedness Inventory (Oldfield, 1971) to assess the dominance of their right or left hand in everyday activities. Results of the quotient were converted into a handedness index (*H*),

(1) $$H=\frac{R-L}{R+L},$$

where *R* is the number of activities on the inventory reported as performed right handed, and *L* is the number of left handed activities. A value of *H* ​= ​1 represents predominantly right handedness, whilst *H* ​= −1 indicates predominantly left handedness. Table 1 provides details of the handedness index and fMRI protocol used in each subject.

**Table 1:** Details of subject’s handedness and protocol used.

Main Session				Reproducibility session		
Subject ID	Handedness Index	Image Resolution/mm	Cycles	Resolution/mm	Cycles	Time between sessions
001	1	1.5	L:12 ​R:10	1.5	12	2y 6m
002	1	1.25	8	1.5	12	5m
003	0.85	1.25	8	1.5	12	5m
004	1	1.25	8	1.5	12	5m
005	1	1.5	12			
006	0.2	1.5	L:12 ​R:10			
007	1	1.5	10			
008	1	1.5	10			
009	1	1.25	8			
010	1	1.25	8			
011	1	1.25	8			
012	1	1.25	8			
013	1	1.25	8			
014	0.6	1.25	8			
015	0.71	1.25	8			
016	1	1.5	L:12 ​R:8			
017	1	1.5	8			
018	0.41	1.5	12			
019	1	1.5	10			
020	1	1.5	12			
021	1	1.5	12			
022	1	1.5	12			

## 3 Data analysis

**Fig. 1:**
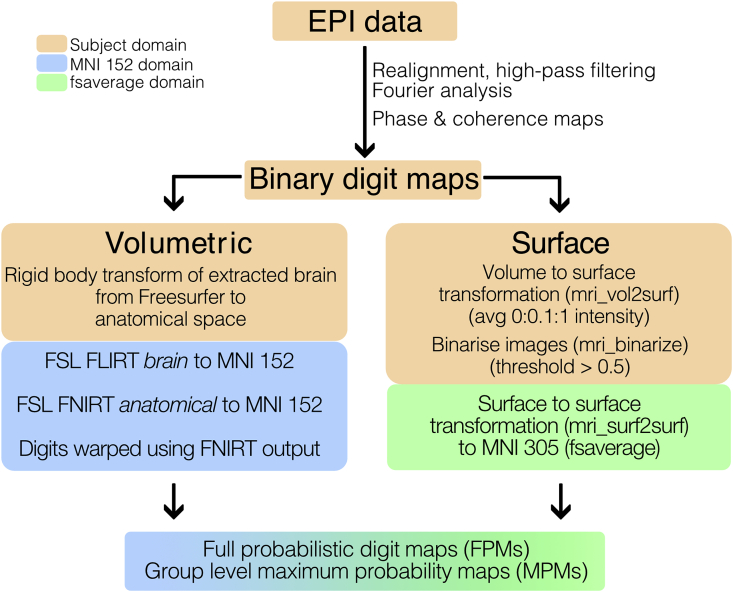
Flowchart of the analysis pipeline from the fMRI data collected using a travelling wave EPI acquisition to the group-level probabilistic atlas of the digits.

The following provides a detailed description of the processing of the functional MRI data to generate the probabilistic atlas. A flowchart summarising the processing pipeline is shown in Fig. 1.

### 3.1 Generation of subject-specific travelling wave maps

The travelling wave fMRI datasets from each individual subject were analysed using mrTools (Gardner et al., 2018). Functional MRI data sets were realigned to the last volume of the data set (reference EPI frame) acquired closest in time to the high-resolution T2∗-weighted dataset. To account for scanner drift and other low-frequency signals, all time-series were high-pass filtered (0.01 ​Hz cut-off) and converted to percent-signal change for subsequent statistical analysis. The forward and backward travelling wave scans for the left and right hand were combined to cancel the haemodynamic delay (Besle et al., 2013). For each voxel, the corresponding time series were Fourier-transformed, with the phase and amplitude of the best-fitting 1/20 ​Hz sine wave computed. In addition, the coherence between the time series and the best-fitting sinusoid were calculated (Engel, 1997; Engel et al., 1994). The phase and coherence statistical maps were then transformed from native functional data acquisition space into the subject’s whole-head anatomical space via a two-step procedure; the reference EPI frame was aligned to the in-plane T2∗-weighted anatomical volume using non-linear alignment to account for any residual distortions in the functional volume (note that image-based shimming limits the field perturbations to <20 ​Hz (Besle et al., 2013; Sanchez Panchuelo et al., 2010; Schluppeck et al., 2018), the in-plane T2∗-weighted anatomical volume was contrast-inverted and linearly aligned with the T1-weighted reference volume. All alignment steps were performed using an iterative, multi-resolution robust estimation method (Nestares and Heeger, 2000) as implemented in mrTools. Cortical segmentations were obtained from the whole head T1-weighted anatomical volume using Freesurfer v5.3.0 (Dale et al., 1999; Fischl et al., 1999a). To ensure accurate segmentation, quality assurance steps were taken. The first step was to check whether all skull and dura were automatically removed during the skull-stripping step; any remnant voxels containing either of these were manually edited. The second step was to check that all white matter regions had been successfully identified, as this process is susceptible to errors associated with B1 inhomogeneities in the anatomical image. Control points were added to regions where Freesurfer returned false negatives and false positives and were removed. All other parameters were set to default.

### 3.2 Subject-specific digit ROI definition

**Fig. 2:**
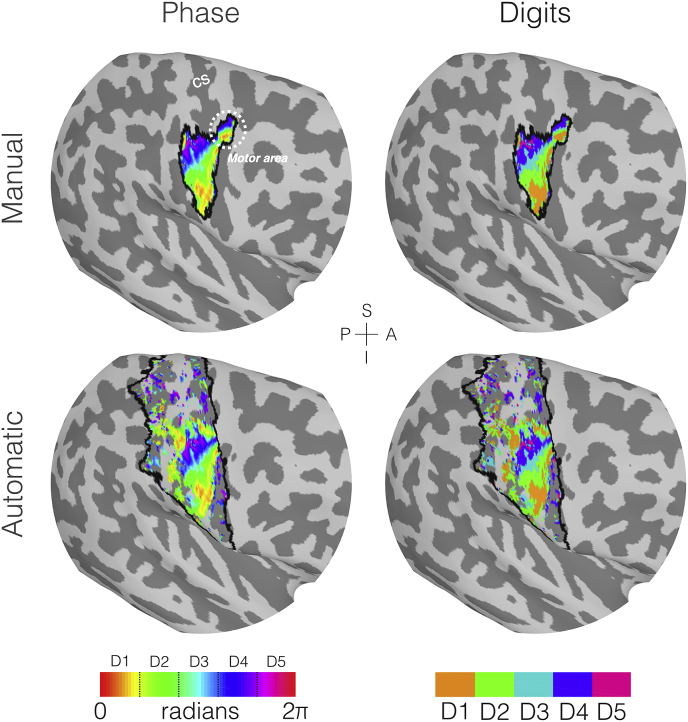
Example of the masking and phase binning process performed on the travelling wave data to generate the digit maps for the left hand (right hemisphere) of a single subject (Subject 003). For visualisation purposes this has been displayed on the subject’s inflated cortical surface. Here light grey patches represent gyri and the darker grey patches the sulci. The central sulcus (CS) is labelled in white in the top left plot for reference. In all plots the mask boundaries are represented by the black lines, with the example of the manual mask on the top row and the automatic mask on the bottom row. Left column: phase maps generated from the Fourier analysis of the EPI data, with only the phase values which survive both the coherence masking and manual/automatic masking displayed. Right column: phase maps binned into the binary representations of the individual digits, note the digit allocation for any given location is mutually exclusive in a single subject using this analysis method. Note that in this subject, we also observe high coherence and phase ordering in the primary motor cortex (M1; highlighted in the dashed circle); an effect which was observed in 50% of subjects.

Once the phase and coherence maps had been transformed into the subject’s anatomical space, two stages of masking were applied to the phase data. The first stage involved statistical masking, based on the coherence maps. Here, the coherence maps were converted to t-values (Besle et al., 2013) and a binary mask of t-values corresponding to p ​< ​0.05 (uncorrected) generated. A second stage of masking of the phase maps was then applied in which two methods were assessed: *manual* and *automatic* masking, as illustrated in Fig. 2. In the *manual* case, the phase data were projected onto the cortical surface representing the midway between the white matter and pial surfaces within mrTools and the subject-specific mask was manually drawn on the surface so as to encompass all vertices whose phase shows an orderly representation of the digits. For the *automatic* approach, the subject specific Freesurfer labels of Brodmann areas 1, 2, 3a and 3b of the hemisphere contralateral to the stimulation were combined to form a mask of the entire somatosensory cortex. For both approaches, the surface-based masks were projected back into the volume space for masking of the volumetric phase data; any voxels between the white matter and pial surfaces that were orthogonal to each vertex of interest in the surface ROI were included in the volumetric mask.

Any voxels of the phase map within the intersection of the statistical and the *automatic* or *manual* masking were then binned to generate subject-specific digit maps. Separate sets of digit ROIs were created using both the *manual* or the *automatic* ROIs. Here, the individual digit ROIs were formed by dividing the phase map into 5 equally spaced bins each of 2π/5 width, with a separate binary image generated for each bin. The binning approach is demonstrated in Fig. 2. Note that the procedure of projecting either the *manual* or *automatic* masks back into volume space restricts these digit maps to the subject’s cortical ribbon, even for the volumetric atlas.

### 3.3 Reproducibility

In four subjects, the reproducibility of the digit somatotopic (phase) maps was assessed across two scan sessions by computing the intersession phase difference for each voxel within the *manual* mask (which comprises voxels specific to the hand area, in contrast to the automated mask which contains voxels within the entire primary somatosensory cortex). Only voxels with coherence equivalent to an uncorrected p ​< ​0.05 across both sessions were considered. To assess reproducibility, we adopt a similar process to that used in (Sanchez Panchuelo et al., 2012). If two phase maps agree with each other, we would expect the distribution of the phase differences between sessions to be both predominantly centred over 0 and unimodal. We tested against this hypothesis using the Raleigh test of uniformity (V-test) within the circular statistics toolbox for MATLAB (Berens, 2015), which tests whether our phase difference distribution can be explained as a uniform distribution across all possible phases (Durand and Greenwood, 1958).

### 3.4 Atlas generation

After the creation of the subject-specific digit ROIs, each of the digit ROI maps were sequentially transformed to both volumetric and surface standard space.

*Volumetric normalisation:* The extracted brain from Freesurfer was rigid-body-transformed to the subject’s anatomical space using FSL’s Linear Image Registration Tool (*FLIRT*; Jenkinson et al., 2002; Jenkinson and Smith, 2001). A two-pass process was used to register the subject’s anatomical volume to the MNI-152 space (2 ​mm isotropic resolution). The first pass involved a linear registration of the extracted brain to the MNI brain using *FLIRT*, and then the resultant transformation was used as an initialisation step for a non-linear warp of the full anatomical (with skull) to the MNI brain (also with skull) using FSL’s *FNIRT*. The resultant warp field was then applied to the registered subject’s digit ROIs, with nearest neighbour interpolation to ensure mutual exclusivity of digit assignments to voxels was maintained.

*Surface projection and normalisation:* The digit ROIs were projected to the subject’s surface space using Freesurfer’s *mri_vol2surf*, using the average of projections at 0%–100% of the distance from the white matter to the pial in steps of 10%. As this process makes the resultant digit maps non-binary, surface maps were rebinarised, with a threshold set at 0.5, which also ensures mutual exclusivity of a single digit to a given vertex. Finally, a surface-to-surface transformation to the MNI-305 (Freesurfer’s *fsaverage* subject) was performed with *mri_surf2surf,* using a nearest-neighbour interpolation.

In both the volumetric and surface cases, the affine transformations from EPI space to subject space and subject space to MNI space were combined to provide a one single transformation of the travelling wave data, thus limiting interpolation effects from the realignment. After normalisation, two classes of probabilistic maps were generated (Wang et al., 2015). Full probabilistic maps (FPMs) are defined for each digit. On these maps, probabilities at each voxel/vertex are defined as the number of subjects for which this particular digit was assigned to this voxel/vertex, divided by the total number of subjects. From these FPMs, we also generate a *digit hand ROI,* which represents where the probability of *any* digit being assigned to a voxel/vertex is higher than 0.5. Here, the digit hand ROI is generated by summing all the FPMs and binarising, with a threshold of 0.5. Maximal probabilistic maps (MPMs) are defined across all digits and assigned to each voxel/vertex within the digit hand ROI to the digit with the highest probability across all digit FPMs. Here *FPM digit ROIs*, binary representations of each digit as defined by FPMs are also created. FPMs and MPMs were generated in both surface and volumetric space with both the *manual* and *automatic* masked data.

### 3.5 Atlas characterisation and validation

In order to quantitatively characterise each atlas, we followed some of the methods previously described to assess visual topography, computing the blurring metric (Fischl et al., 1999b) and central tendency (Eickhoff et al., 2007).

The blurring metric provides a measure of how well ROIs from individual subjects overlap in a standard space. If a spatially normalised digit ROI from subject *k* is considered as a set $S_{k\;}\left(k\;=\;1\;\text{to}\;n\right)$, where each element of the set are the voxel/vertices where a ROI exists, then the blurring metric, *B*, for a given digit is

(2) $$B=\;100\frac{\left|\cup_{k=1}^{n}S_{k}\right|-\;\frac{1}{n}\sum_{k=1}^{n}\left|S_{k}\right|}{\frac{1}{n}\sum_{k=1}^{n}\left|S_{k}\right|},$$

where $\left|S_{k}\right|$ is the set cardinality, or number of elements in a set. Put simply, this is the percentage difference between total number of unique voxels/vertices attributed to at least one digit in any subject and the average spatial extent of a digit ROI across all subjects. For example, if a digit area was on average 1 ​cm^2^ across subjects, a blurring metric of 800 would mean the group-level FPM has a surface area of 8 ​cm^2^. A perfect overlap would return a blurring metric of 0.

The central tendency quantifies how much a single subject’s digit ROI overlaps with high probability areas of the corresponding group-level FPM. If we assume the FPM is represented as a $1\times n_{locations}$vector f and a subject’s binary digit ROI is a vector of the same dimensions, d, the central tendency P between the *i*th FPM and *j*th digit ROI is

(3) $$P_{ij}=\frac{F<f_{i}\circ d_{j}>}{F<f_{i}>},$$

where ∘ is the Hadamard product and F⟨⋅⟩ is a function which calculates the average of all absolute non-zero values within the triangle brackets. Fig. 7B has a cartoon diagram of some examples of how the central tendency score would change depending on where a digit ROI falls across an FPM. A value of P=1 implies perfect overlap; a value above 1 means it resides more centrally (i.e. over areas of higher probability); below 1 suggests the digit overlaps with the periphery of the FPM. The unbiased, average central tendency across all subjects (for each digit/FPM combination) was calculated using a leave-one-out approach. In a single iteration, the FPMs were generated with 21 subjects and tested on the remaining subject. We also applied the leave-one-out and central tendency approach with the MPMs. For two binary vectors, the central tendency represents the ratio between the extent of the overlap between two vectors and the magnitude of the test vector, ***f***. Note that the central tendency will never exceed 1 when comparing two binary maps, so direct comparisons between FPM and MPM central tendency scores cannot be made. In both cases however, we would expect the central tendency be maximal for the corresponding digit in the atlas when compared to the others. We expect that, after comparing all leave-one-out permutations, the central tendency score for a given FPM and its respective digit ROI to be higher than for any other digit ROI ($P_{i=j}>P_{i\neq j}$). In doing so this would imply there is a preferential organisation of the digit in question which is largely robust across subjects not included in the atlas.

### 3.6 Anatomical variability

Since the probabilistic atlas of the somatotopic map conveys both functional and structural variability, we sought to investigate how much structural variability plays a part in the functional results. To assess this, we compared the anatomical variability in the somatosensory cortex to the anatomical variability within probabilistic atlases of retinotopy which are formulated using similar methodology (Wang et al., 2015). To assess the mutual alignment of subjects’ anatomy, maps of the mean and variance of the gyral and sulcal convexity across subjects was computed in standard surface space. The mean curvature and variance across the digit hand ROI was computed and compared to that exhibited in the primary and secondary visual cortex (V1, V2) as defined in Freesurfer.

### 3.7 Data availability

Spatially normalised digit ROI maps from the 22 individual subjects and group-level FPM/MPM atlases are available at https://github.com/georgeoneill/digitAtlas. Code to generate the group-level atlases is also available on the repository. Raw data and processing scripts are available on request – please contact either STF or RMSP.

## 4 Results

All participants were right handed as confirmed by the Edinburgh Handedness Inventory, Table 1 provides the handedness index for each individual. Handedness indices ranged from 0.2 to 1, with a mean of 0.89 ​± ​0.05 (standard error); but the majority of subjects had a handedness index of 1 (17 out of 22).

Fig. 2 illustrates the travelling wave fMRI maps on a single example subject (Subject 003). Analysis reveals cortical areas with high digit specificity located on the posterior bank of the central sulcus and the postcentral gyrus, corresponding to the contralateral primary somatosensory cortex (S1). Phase maps (Fig. 2) show an orderly pattern ranging from low (digit 1) to high (digit 5) values following the main inferior/superior direction along the central sulcus (and lateral/medial direction given the orientation of the sulcus in the coronal plane). In this particular subject, we also observed high coherence and phase ordering in the primary motor cortex (M1; highlighted in Fig. 2 as a dashed circle); an effect which was observed in 50% of subjects (11). Note that the use of the *automatic* masking method will exclude this area of activation as it occurs in Brodmann area 4.

**Fig. 3:**
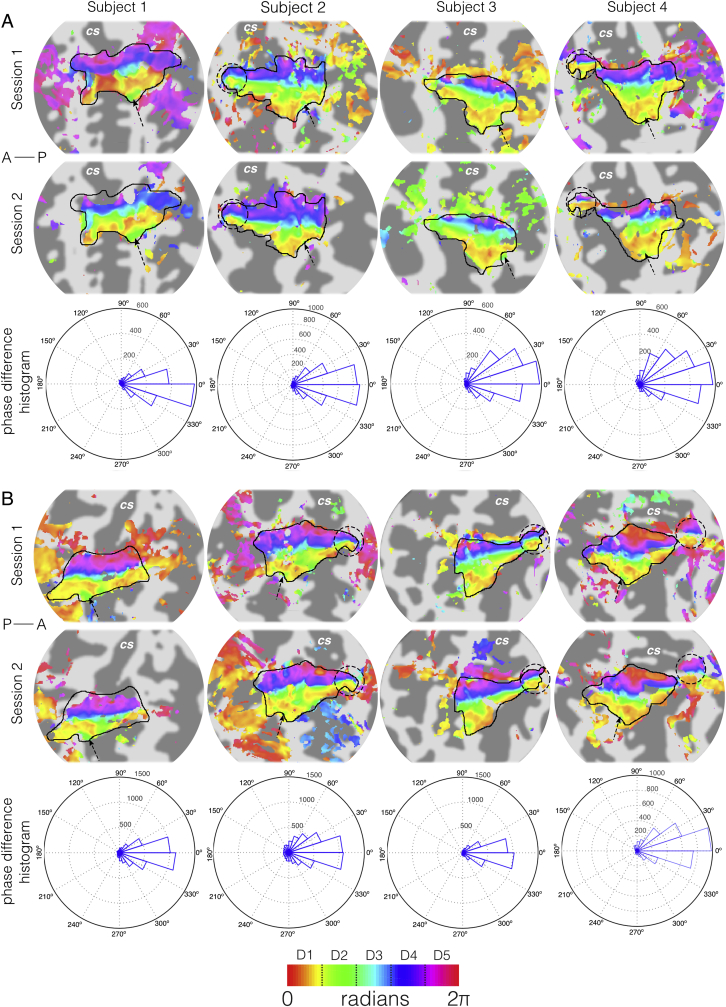
Reproducibility of digit somatotopic maps for (A) right hand (left hemisphere) and (B) left hand (right hemisphere). Phase activation maps (displayed above a coherence value of 0.3 – as used in Da Rocha Amaral et al., 2020) from data acquired in two separate scanning sessions onto flattened representations of the contralateral central sulcus. Session 1 (first row) data acquired at 1.25 ​mm isotropic resolution for all subjects except Subject 1 (1.5 ​mm isotropic resolution). Session 2 (second row) data acquired at 1.5 ​mm isotropic resolution. Dark grey, areas of negative curvature (sulci); light grey, areas of positive curvature (gyri). An orderly representation of the digits is seen in the posterior bank of the central sulcus (CS) and postcentral gyrus, corresponding to S1. The black outline shows the manual delineation of the cortical surface for digits. In some subjects’ extra features are seen: a full representation of the digits in the primary motor cortex (dashed circles) and/or a secondary area for D2 which surrounds D1 (dashed arrow). Third row: Histograms of voxel-wise phase differences between travelling-wave scan sessions showing phase difference values centred around 0 indicating high similarity between scan sessions.

Phase maps are highly reproducible across subjects, as illustrated here by the results from the four subjects who participated in two scan sessions for both the left and right hand (Fig. 3). The distribution of the inter-session phase difference values in the *manual* hand ROI mask for each subject was significantly non-uniform and distributed around 0 (V test, V ​= ​2116 ​± ​596 and 3090 ​± ​262 [mean ​± ​standard deviation across subjects] for both the right hand and left hand digits respectively, p ​< ​10^−16^ for all subjects). This underscores the high reproducibility of the phase maps between scanning sessions, even when the spatial resolution of the fMRI data within a subject differed (1.25 ​mm and 1.5 ​mm isotropic voxels).

**Fig. 4:**
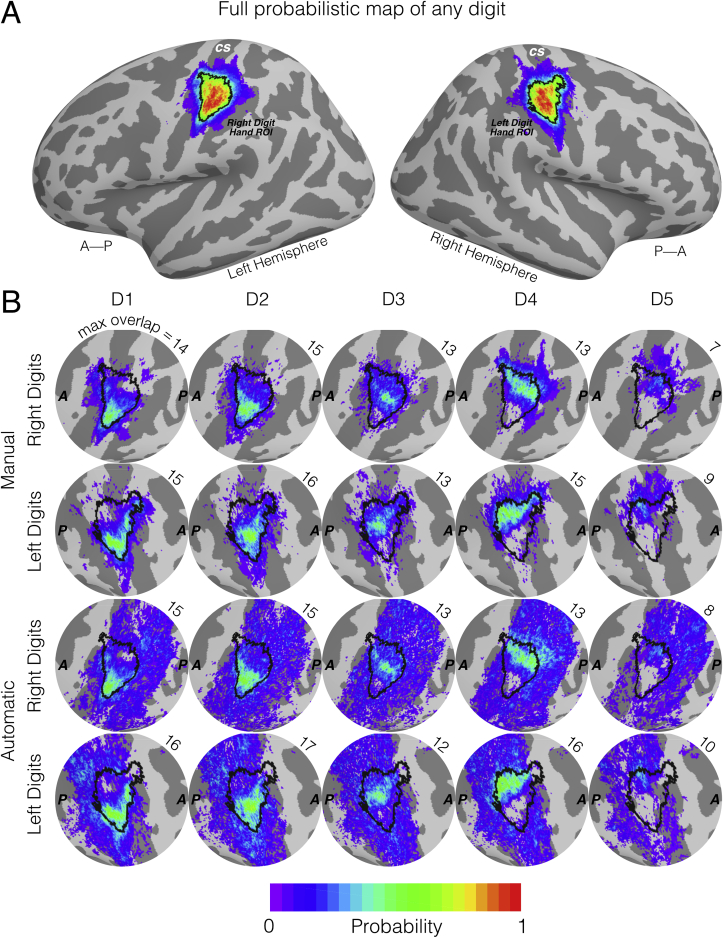
Full Probabilistic Mapping (FPM) for a surface-based atlas of the digit areas of the hand on an inflated cortical surface. Dark grey cortical regions represent sulci, whilst light grey represent gyri. A) The summation of all 5 digits for right and left hands (left and right hemispheres) to show the probability of functional activation of any digit of the hand, showing the largest areas of highest probability in the posterior bank of the central sulcus and postcentral gyrus. The black boundaries represent the 50% probability threshold with the enclosed ROI referred to as the digit hand ROI. B) Zoomed-in representations of the left and right central sulci, with the corresponding digit hand ROI overlaid for reference. Each sub panel represents the FPMs for each individual digit of the hand for both the manual and automatic masking. The number in the top right corner of each sub panel shows the maximal number of overlapping subjects across voxels. The individual subject maps, combined to form the FPMs, are available to view in the supplementary material and available to download at http://github.com/georgeoneill/digitAtlas.

Fig. 4A shows the surface full probability map (FPM) of any digit being represented (i.e. the sum of all FPMs), for both the left and right hands, when *manual* masking was applied. We observe the highest probabilities in the posterior bank of the central sulcus (CS) and postcentral gyrus corresponding to primary somatosensory cortex (S1). In both the left- and right-hand FPM, many voxels show probability values of 1 (i.e. 100% overlap across all 22 subjects). Fig. 4B shows surface FPMs for the individual digits of the left and right hands for both the *automatic* and *manual* masks. Both methods can be seen to yield similar maps with the FPMs from D1 to D5 following the expected lateral-to-medial organisation along the central sulcus. Note that the *automatic* masking method allows for digit assignment to any region within (probabilistic) S1 and this is reflected by the larger spatial extent of low probability values compared to the *manual* masking. In the top right corner of each FPM plot, the maximal number of subjects (of the 22 included in this probabilistic map) with overlap for a given digit is provided. Here we see that the peak overlap is similar across both the *manual* and *automatic* masking, with a maximum of 17 for left D2 (*automatic* masking) and minimum of 7 for right D5 (*manual* masking). For the volumetric-based pipeline (Supplementary Material), we observe a similar digit organisation, but with lower peak overlap values across subjects for all digits (maximum: left D2, *manual* ​= ​15; minimum: right D5, *manual* ​= ​5). The individual subject maps, combined to form the FPMs, are available to view in the Supplementary Material. To simplify the results, we shall from this point onwards only discuss the results of the *manual* masked data, with *automatic* masked results provided in Supplementary Material.

**Fig. 5:**
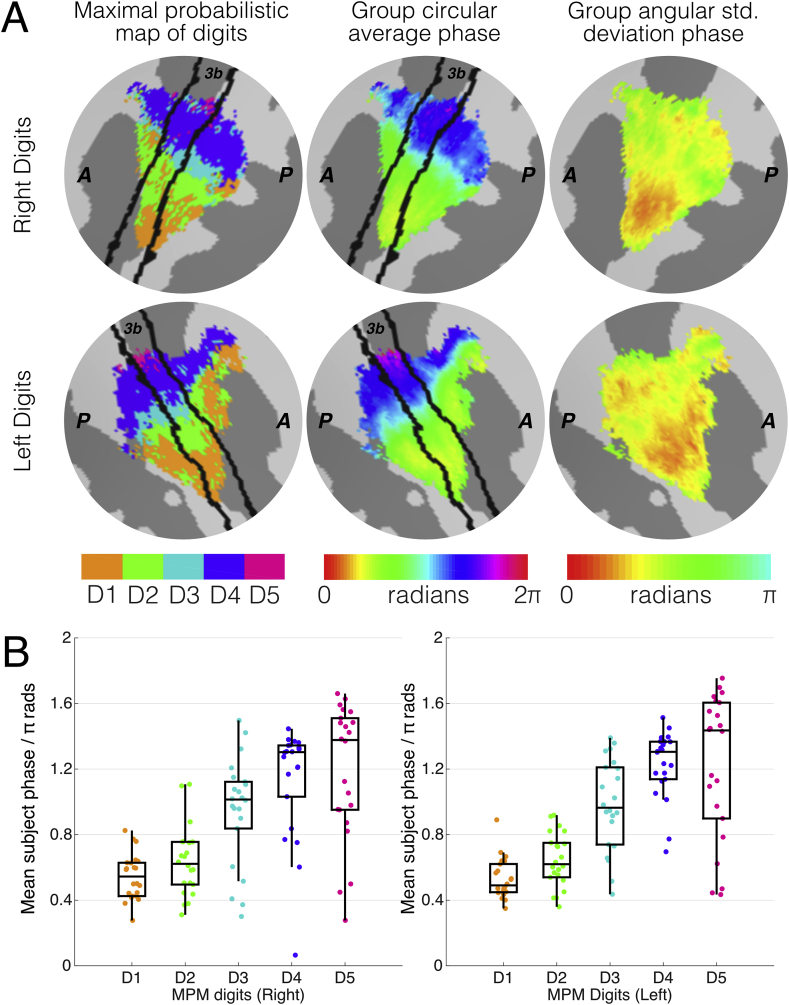
The relationship of the Maximal Probability Maps (MPM) to the group-level phase results. A) Left column: the MPMs for the digit areas based on the winner-takes-all approach, showing the lateral-medial organisation of the digits. Overlaid is Freesurfers Brodmann area 3b label for anatomical reference (black lines). Middle column: the group circular averaged phase maps (in radians), masked to contain only the regions represented by the MPMs. Right column: circular standard deviation of the phases across subjects (in radians), showing lower variabillity in the left digits (averaging 1.08 radians across the ROI) compared to the right digits (1.22 radians). B) The relationship between the MPM ROIs and each subjects’ average phase, each point represents the mean phase for a subject over that ROI in MNI space. Overlaid is the box and whisker plot showing the median, interquartile range, and minimum and maximum values. Grey horizontal lines represent the boundaries between digit allocations. The median of the subject distributions for digits 2, 3 and 4, but not digits 1 and 5, fall within the expected boundaries.

Fig. 5A(left column) shows the maximum probability maps (MPMs) for the right and left digits (surface analysis, *manual* masking), again showing the lateral-to-medial organisation of the digits, and its relation to *fsaverage*’s Brodmann area 3b label. Fig. 5A shows the group circular-average (middle column) and associated standard deviation (right column) of the phase maps, masked to show only those locations found in the MPMs. It can be seen that the progression of low to high phase values are organised in a similar fashion to the MPMs. It is important to note that simply binning the group average phase map to generate group-level ROIs would not give the same result as the MPM approach. There is a negligible representation of both D1 and D5 phases in the group circular average phase maps. This is further illustrated in Fig. 5B which plots the average phase value for each subject (transformed to *fsaverage* space) within each MPM digit ROI. Whilst a monotonic increase is seen in the median values of phase with each digit, only the median phase of digits 2, 3 and 4 falls within the expected ranges (e.g. D2 in single subject is defined by a phase between 0.4π and 0.8π, and this is reflected in the group median). The lack of phase definition for digits 1 and 5 reflects the information lost by simply averaging phases across the group.

**Fig. 6:**
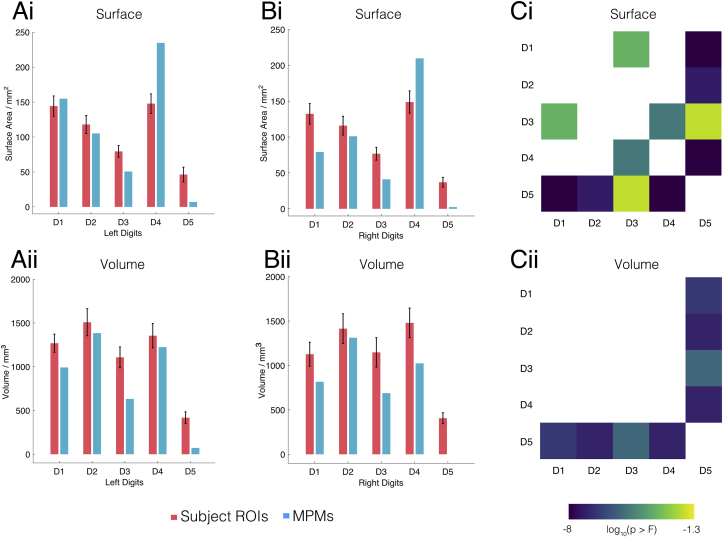
Digit ROI size results, surface-based results are shown on the top row and volume-based results on the bottom. A) A comparison of the subject left digit ROI surface areas when projected to MNI spaces (pink bars) with the resultant MPM ROIs (blue bars), error bars (black) represent the standard error of the mean. B) The same as panel A but for the right digits. C) The results of the post-hoc pairwise comparisons of the spatial extent of digit ROIs. The matrices contain log-transformed p-values of the null hypothesis that the labelling of the digits doesn’t affect the size of the digit ROI. Note that a value of −1.3 corresponds to p ​= ​0.05, meaning that the size difference between two specific digits is significant. In all cases, D5 is significantly smaller in representation than the rest of digits.

Fig. 6 shows the average surface area of the individual subject digit ROIs and MPMs after normalisation to the standard surface (Fig. 6Ai and 6Bi) and average volume (Fig. 6Aii and 6Bii) in volumetric space. Note that surface ROIs are projected to Freesurfer’s *fsaverage* white matter surface, and surface areas will slightly vary depending on the cortical surface of choice. Pink bars represent the group averaged spatial extent of each digit ROI whilst the blue bar is the spatial extent of the corresponding MPM. An ANOVA with digit allocation and hand as factors revealed a statistically significant effect of digit allocation on ROI surface area (surface: F(4,210) ​= ​26.32, p ​= ​1.06 ​× ​10^−17^; volume: F(4,210) ​= ​19.90, p ​= ​6.60 ​× ​10^−14^), but no effect of hand (surface: F(1,210) ​= ​0.41, p ​= ​0.52; volume: F(1,210) ​= ​0.03, p ​= ​0.86) or interaction between the digit allocation and hand (surface: F(4,210) ​= ​0.1, p ​= ​0.98; volume: F(4,210) ​= ​0.31, p ​= ​0.87). There was no significant difference in the surface area and volume associated with the individual subject digits between left and right, post-hoc tests were performed on data from both hands combined. Fig. 6Ci and 6Cii show the p-values (Bonferroni corrected) for all pairwise comparisons between digit ROI surface areas for surface and volume analysis respectively. In summary, we observe that the size of D5 is considerably smaller than all other digits in both the surface and volumetric maps, whilst D3 is smaller than D1 and D4 only in surface space. The MPM ROI sizes (blue bars) follow a similar trend as the average digit ROI sizes, particularly for the left digits. However, we observe in the surface-based analysis that the size of D4 in the MPM is notably larger than the group average size, whilst D5 is markedly smaller in all cases.

**Fig. 7:**
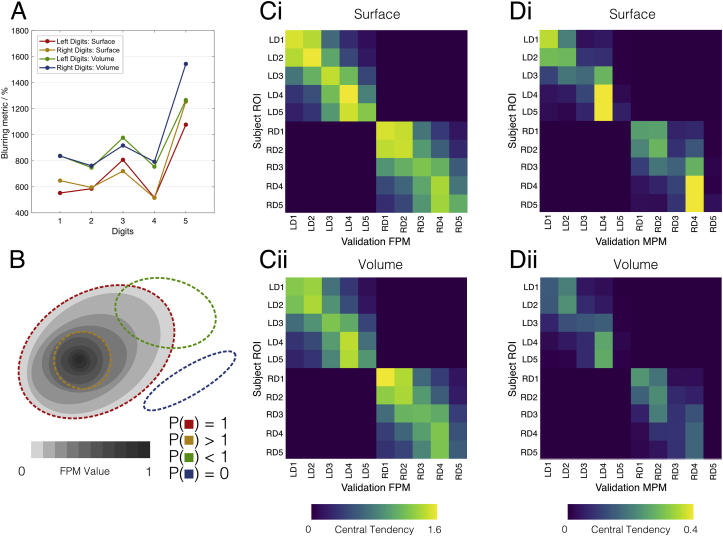
Characterisation and validation results of the manual probabilitic maps in both surface space and volume space (bottom). A) Blurring metric for the digit ROIs after spatial normalisation; lower scores indicate a better overlap of the ROIs across normalised subject maps. ROIs in surface space yield lower blurring scores compared to their volumetric counterparts. B) A cartoon diagram showing how the central tendency score, P, is affected by how a candidate ROI (dashed ellipses) overlaps an FPM. Here the scores are larger than one if an ROI is focalised over larger values of the MPM, a score of unity is achieved if it completely overlaps all non-zero values of the MPM and it tends to zero the less it coincides. C) Central tendency scores from FPMs generated using the leave-one-out method, with the average scores from all 22 leave-one-out permutations shown. There is a strong diagonal element to these matrices, with the central tendency scores being highest along the diagonal for a given ROI in 8/10 digits for the surface ROIs and 7/10 for the volumetric ROIs. D) The average central tendency score for MPMs generated using the leave-one-out method.

Validation results are provided in Fig. 7. First, the blurring metric (Fig. 7A) shows the highest overlap (i.e. lowest blurring metric) in D4, followed by D2, D1, D3, with D5 displaying a much higher blurring metric compared with the other digits. It can also be seen that the blurring metric for the surface maps is lower than for their volumetric counterparts, which conforms with previous studies (Fischl et al., 1999b; Wang et al., 2015). Comparing digits within the same atlas, we observe that there is a strong anti-correlation between average digit ROI size and blurring metric (surface atlas: r ​= ​−0.934, p ​= ​7.3 ​× ​10^−5^; volume atlas: r ​= ​−0.956, p ​= ​1.7 ​× ​10^−5^). Leave-one-out validation results are depicted as matrices of average central tendency across the 22 leave-one-out iterations of the FPMs and MPMs (see methods). For the FPMs (Fig. 7B), it can be seen that, in most cases, the diagonal element of the central tendency matrices is the largest in each row, implying that the FPMs built from 21 subjects show high overlap with the ROI locations of a novel subject. This is the case for all digits except D5 in both hands and both in the surface and volume representations. For D5 of both hands, the central tendency for D4 is the dominant value, suggesting that the FPM for D4 regularly overlaps with a subject’s D5. In the volumetric case, a similar effect is observed for left D1 and D2: the central tendency of D1 is higher in the D2 than the D1 FPM. When assessing the leave-one-out results for the MPMs (Fig. 7C), recall that we cannot compare central tendency scores between the MPMs and FPMs due to the fact the MPMs are binary images, but can compare where the distribution of values across the digits lay. To that end, we see that fewer maximal central tendency values lie along the diagonal for the MPM. In other words, fewer digit ROIs show the highest overlap with the corresponding MPM ROI. Rather than 8/10 digits in the FPM atlas (Fig. 7B) showing the highest central tendency value for each row, in Fig. 7C we see that this is only the case for 4/10 digits (surface case, Fig. 7Ci) and 5/10 (volume case, Fig. 7Cii) respectively. Here in particular it becomes apparent that, since in the MPMs, the representation of D4 is considerably larger than that of other digits (see Fig. 6), it is more likely to overlap with subjects’ D3 and D5 ROIs. Note also, that due to the almost negligible size of D5 in the MPMs, the central tendency score of any digit with D5 is close to zero.

**Fig. 8:**
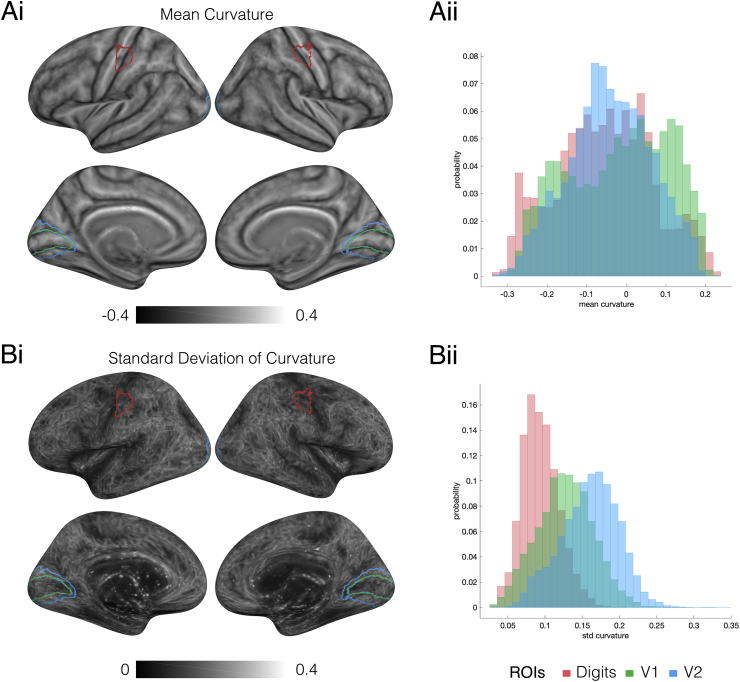
Results of the anatomical variability analysis in the surface domain. Ai) Maps of average surface curvature across subjects after transformation to the MNI-305 space. The ROIs for the digit hand area, V1 and V2 are shown on the surfaces in red, green and blue respectively. Aii) Nomalised histograms show the distributions of average curvature across vertices of the three ROIs. The gamut of curvature values is similar across the three ROIs. B) Maps of curvature variability, as represented by standard deviation across subjects. Bii) Normalised histograms of curvature variability across vertices of the three ROIs. Anatomical differences within the digit hand area in S1 are notably lower than in V1 and V2 areas.

Fig. 8 shows the results of the anatomical variability analysis. Fig. 8Ai shows the mean curvature across subjects over the entire left and right cortical surfaces and Fig. 8Bi shows the corresponding standard deviation. Fig. 8Aii and 8Bii show the distributions of mean curvature and standard deviation in three test ROIs: the digit hand area (red), V1 (green) and V2 (blue). It can be seen that the ranges of average curvature values are similar across all three ROIs, but that the digit hand ROI has reduced curvature variability (median standard deviation ​= ​0.091) compared to V1 (median standard deviation ​= ​0.127) and V2 (median standard deviation ​= ​0.163). These results show that anatomical variability is less of a factor in the somatotopic maps than for retinotopic maps formulated in a similar fashion.

## 5 Discussion

Here, we present a probabilistic volume- and surface-based atlas of digit somatotopy from functional ultra-high field MRI data. These maps are derived from finger dominance and are shown to be highly reproducible across multiple measurements for a given subject (Fig. 3). We compute full probabilistic maps (FPMs), showing that the lateral-to-medial organisation of digits 1–5 is present at the group level; with high levels of spatial agreement across subjects for digits 1–4 and a weak overlap for digit 5 (Fig. 4). We attempt a hard allocation scheme – the maximal probability map (MPM), which while retaining the stereotypical organisation of the digits is not entirely representative of the underlying maps (Fig. 5). The group representation of the digits are not separated by clear-cut boundaries, but rather has a continuum across digits. We show clear group-level digit 2, 3 and 4 maps, whilst digit 1 often overlaps with digit 2 (Fig. 7). We compare two methods for masking the data, a *manual* defined ROI and a less constrained *automatic* masking process. We find the surface-based atlas gives a better resolved map of the digits than the volumetric counterpart, and the *manual* masking returns less blurred digit maps than the *automatic* method.

### 5.1 The effect of surface versus volume normalisation on FPMs

In the FPM, we observe for both the left and right digits a clearly defined ‘digit hand ROI’, with a 50% or greater probability of any digit being represented in both surface space (Fig. 4) and volume space (Supplementary Material). Furthermore, we observe regions with 100% overlap across subjects in surface space and 95% in volume space. Within the ‘digit hand ROI’, the FPM of individual digits (D1-D5) shows a clear lateral-medial organization in contralateral S1 (Fig. 4). We also observe that individual digit overlap is generally greater in the surface atlas than in the volume atlas (Table S1 in Supplementary Material). This may be due to at least two factors: 1) surface-based normalisation provides better data registration to a standard template (here *fsaverage*), and therefore offers better overlapping group data (Aquino et al., 2019; Fischl et al., 2008, 1999b; Lerch et al., 2017; Wang et al., 2015), which is also reflected in this study from lower blurring metrics for the surface-based digit maps compared to their volumetric counterparts (Fig. 7A); 2) the cortical surface offers a finer spatial resolution than the 2 ​mm volumetric brain – a subject averaged inter-vertex distance of 0.8 ​± ​0.1 ​mm over the digit hand area – allowing for finer sampling of the data for investigating overlapping representations.

### 5.2 The effect of automatic versus manual masking on digit dominance

We also assessed how masking of the travelling wave data affects the digit maps, either using a *manual* (hand-drawn ROI based on an orderly and coherent phase map) or *automatic* (pooling of ROIs of Brodmann areas 1, 2, 3a, 3b) method. Fig. 4B shows both mask types produce comparable areas of higher probability, however *automatic* masking yields a larger spatial extent. This can be seen for a single subject (Fig. 2), where the *manual* ROI is mostly enclosed within the *automatic* masked area, leading to similar phase and binned digit maps within that core region. Both masking methods have advantages and drawbacks; *manual* masking leads to a focal area of activation which is biased to the areas of orderly representation but it is more subjective. The *automatic* method is less constrained and more trivial to reproduce, but has disadvantages; first, the lack of specificity to body site means that the spatial extent of each digit in the MPMs span almost the entire primary somatosensory cortex (large areas of deep blue and purple (low probability) in Fig. 4B). The low probabilities of the digits FPMs individually in these areas should not be a problem, but the effect compounds when summing the maps, resulting in a digit hand ROI which spans from the Sylvian to central fissures (Supplementary Material). These diffuse functional areas also lead to comparatively worse separation of the digits compared to using the *manual* masking; our leave-one-out analysis shows a reduction in selective preference, with at best only 5/10 digits correctly attaining the highest central tendency scores (Supplementary Material). Second, a rigid definition of S1 means that some functional areas that are consistently activated are overlooked such as the section in primary motor cortex (M1), which has a full coherent representation of digits in only half of subjects (Figs. 2 and 3). This M1 map has been observed in previous high spatial resolution studies (Besle et al., 2014, 2013; Sanchez Panchuelo et al., 2010), and we suggest that this reflects genuine localisation of function, rather than an unfolding artefact, especially given the smaller spatial extent in M1 contains the entire range of phase values. However, future studies should investigate this further to confirm whether this is a genuine somatosensory process or the result of a motor response when reacting to the somatosensory stimulation.

### 5.3 Digit allocation in MPMs

Maximal probabilistic mapping (MPMs) allow us to generate sharp boundaries between digits at the group level (Fig. 5, with volumetric FPMs and MPMs shown in Supplementary Material). Digits D1, D2, and D4 show a larger representation than D3 and D5. D5 representation is particularly small, with the size of the MPM D5 area being notably smaller than the group mean size of the D5 ROI across subjects (Fig. 6), while D4 is considerably larger. Comparing to previous studies, there was no significant difference in the surface area and volume associated with the individual subject digits between the left and right hand, which agrees with a recent investigation into the functional organisation of dominant and non-dominant digits (Schweisfurth et al., 2018). The finding of large D4 and small D5 in the MPM can be explained by both a low spatial overlap for D5 across subjects and a large overlap for D4. For example, in the right hand, the maximal probability of a vertex representing D5 is 0.32, whist for D4 this rises to 0.59. Consequently, the winner-takes-all digit allocation in an MPM means that higher overlap for D4 allows it to expand into D5 territory. There is also the possibility that D5 should extend more medially than it does in the MPM, but due to low overlap across subjects, it does not satisfy the classification for digit assignment (i.e. probability of *any* digit being allocated at a given location exceeding 50%). Consistent with this interpretation, the group-level phase map (Fig. 5) show a progression of phase in close keeping with the MPMs for digits D2, D3 and D4 but not for D1 and D5. In a large number of subjects (n ​= ​14 and n ​= ​12 for right and left hand somatotopy respectively), there is an additional region responding preferentially to D2 inferior to the representation for D1 (Fig. 3, dashed arrows in subjects 1 and 4’s maps), implying that D2’s functional digit dominance ‘sandwiches’ D1. A previous fingertip somatotopy study (Besle et al., 2014) using an event-related design has shown that these inferior D2 areas are broadly activated by all digits but with highest statistical significance for D1 or D2. It is this ambiguity between dominance of D1 and D2 which we believe drives the higher-than-expected values of phase in those areas. Additionally, it can be seen in Fig. 7B and C that the central tendency scores for D1 and D2 are very similar; consistent with a high degree of overlap between D1 and D2 representations. Despite the oversimplification of the winner-takes-all digit representations made by the MPM, this is distinct to a simple binning of the group-averaged phase maps, as shown in Fig. 5B, where the MPM ROIs do not directly correspond with the group-average phase map. For D5, this is illustrated by the small size of D5 representation and the large spatial variability (poor overlap, see high blurring metric values in Fig. 7) across subjects. For D1, this phase misalignment is a more curious result given its good overlap and large spatial extent. We believe that the larger phase values in the group-level D1 ROI are due to the superposition of D1 and D2 receptive fields.

### 5.4 FPM and MPM atlas validity

Fig. 7 shows the results of quantifying how well subjects are aligned to each other and how generalizable the atlas is for a novel subject. The blurring metric shows that the surface-based atlas has overall less variability across individual subject maps than the volumetric counterpart, in line with previous observations (Fischl et al., 1999b; Wang et al., 2015). D5 has a considerably higher blurring metric compared to the rest of the digits, it is difficult to reliably align an area which is on average 0.5 ​cm^2^ across a cohort. We also observe a similar effect with D3 (smaller ROI area, lower overlap, higher blurring metric). The leave-one-out validation results (Fig. 7B and C) show in most cases that the diagonal element of the central tendency matrices is the largest in each row, implying that FPMs generated from 21 subjects show high overlap with the ROI locations of a novel 22nd subject. This is the case for all digits other than D5 in both hands, where the central tendency for D4 is the dominant value. We believe this may be the result of higher probability values in the D4 FPM compared to the D5 FPM generating a bias favouring D4. Here, it is possible to imagine a case where even though a region may overlap the peak of the D5 FPM region, the higher probabilities of distal D4 FPM may contribute more to the central tendency scores. This bias could be addressed by scaling the probabilistic maps into likelihood maps, where peak probabilities are scaled to be equal to 1. However, MPMs cannot be generated from scaled FPM maps, as in some voxels or vertices there would be combined probabilities of any of the digits being stimulated exceeding one, and the statistical rigour of the winner-takes-all approach would be compromised. In the volumetric case, we also find that a novel left D1 corresponds better with the left D2 FPM than the left D1 FPM. This may be due to volumetric spatial normalisation blurring the aforementioned ‘sandwiching’ effect of D2 around D1. When assessing leave-one-out results for the MPMs (Fig. 7C), we see that fewer candidate digits correspond with the ‘correct’ digit in the atlas compared to the FPM counterparts.

### 5.5 Structural versus functional variability

The probabilistic atlas can be influenced not only by the inter-subject variability of functional organization, but also by morphological variation across the somatosensory cortex of individual subjects (Fig. 8). Structural variability can be in terms of folding patterns (Ono et al., 1990) or more specific micro-architectonic features such as the density, size, orientation and shape of cells and myelin sheaths. For example, within S1, it has been shown that there is high inter-subject variability of the cytoarchitectonic boundaries between Brodmann areas (3a, 3b, 1 and 2; Geyer et al., 2000). We show that for the S1 hand area (ignoring boundaries between different primary Brodmann areas 3a, 3b, 1 and 2), the anatomical variability due to gyral and sulcal convexity is relatively minimal, as compared to that in the visual cortex (Fig. 8Bii). This result suggests that the inter-subject variability influencing the probabilistic maps can largely be attributed to variations in functional topography rather than anatomical misalignment. This result, taken together with the good alignment of the functional hand area ROI across subjects, is in line with findings from a recent study (Germann et al., 2019) which shows that anatomical morphometric subdivides the central sulcus in distinct sulcal segments relating to representations of distinct body parts, where the representation of the hand digits spans one of these segments. The finer representation of the individual digits within the hand area is more variable across subjects. Given that there are no sulcal landmarks to subdivide the hand area, and that the surface registration step aligns cortical folding patterns based on cortical curvature, we cannot exclude the possibility that some of the inter-subject variability of the individual digits probabilistic maps is due to anatomical variability of the micro-architectonic features. It has been shown that cortical cyto- and myelo-architectonic features are more strongly related to cortical function than cortical folding patterns (Amunts et al., 2007). Recent studies (Glasser et al., 2016; Kuehn et al., 2017) using myeloarchitectonic mapping techniques have shown correlation of anatomical subdivisions within the S1 to functional cortical fields (defined by the topography to specific body sites). Glasser et al. (2016) defined five structural and functional subdivisions (face, eye, upper limb, trunk and lower limb) within S1. Kuehn et al. (2017) also found that the hand and face representation areas correspond to distinct structural parcellations, with a reduction of myelin found between the two representations. Given the close link between myelination and function, it is therefore possible that by using emerging multi-contrast multi-scale surface registration techniques that include T1-maps to improve cortical alignment (Tardif et al., 2015), the variance of the probabilistic functional maps may be reduced.

### 5.6 On the use of finger dominance and travelling wave paradigms

A potential limitation of our study is that Fourier analysis of the travelling wave data results in digit dominance maps, rather than maps of the complete cortical representation of each digit. Fourier analysis generates maps based on the phase of the BOLD response, with a given phase range assigned to a unique stimulation location; hence cortical areas that respond to multiple digits will only be assigned to the single most dominant digit. In a previous study, we localised BOLD responses to an event-related design and showed there was spatial agreement between activation peaks and digit dominance maps generated using a Fourier approach (Besle et al., 2014, 2013). Further, it should be noted that it is possible to extract digit overlap information from travelling wave paradigms should it be desired, with a recent study directly comparing Fourier analysis to an iterated Multigrid Priors analysis (iMP; Da Rocha Amaral et al., 2007). This showed that there was spatial agreement between the methods, but crucially that iMP could also estimate digit overlap (Da Rocha Amaral et al., 2020). Furthermore, a study by Puckett et al. (2020) applied a Bayesian variant of population receptive field mapping (Zeidman et al., 2018) to estimate receptive field size and centre of mass in S1, and showed digit preference and overlap (Puckett et al., 2020). These recent advances in extracting digit size and location from travelling wave data will prove useful in future, as travelling wave designs are much faster at digit mapping than event related designs (Besle et al., 2013). The shorter acquisition time (by a factor of 3–4) also makes travelling wave paradigms more suitable to study clinical populations, and compare results to a healthy subject probabilistic map.

### 5.7 Applications of the atlases

The potential for these atlases is substantial. An immediate application is when the explicit localisation of the hand and digit areas is not known or difficult; either due to a time/resources constraint in localising the digits, or from an inability to localise the area due to injury. In order to localise the cortical representation of a given digit, one may consider using a weighted combination of the given digit dominance atlas and the adjacent digit/s dominance atlas. The atlases should be particularly useful for targeting areas of interest in other functional imaging modalities. For example, in electro/magnetoencephalography (E/MEG) studies, where the spatial precision is less than that of fMRI, the probabilistic somatosensory atlas can be used to validate localisation of source reconstructed activity, or perhaps as a ROI prior in modelling or connectivity analyses. In the Appendix, we demonstrate this application of the probabilistic atlas with MEG data, where it is used to confirm that a specific spectral sub-band of rhythmic neural activity originates from the digit-specific locations in the cortex response to somatosensory stimulation.

## 6 Conclusion

The probabilistic maps generated here provide a method to define the likelihood of a given coordinate being associated with a particular functionally defined digit over a population of subjects. In the future, this can be used to infer the localisation of the digits in the primary somatosensory cortex of any newly acquired data set, including at an individual subject level. The cross-validation analysis shows that the FPM is a useful predictor for individual digit S1 representations in novel subjects who did not contribute to the atlas creation, albeit with a large overlap between D1 and D2. In contrast, the MPM was not generally predictive of single subject digit ROIs for novel subjects. However, there are cases where the MPM may be preferred, for example where one needs hard boundaries between digit ROIs. The group-level MPM and FPM atlases as well as spatially normalised individual subject maps have been made available at https://github.com/georgeoneill/digitAtlas and will periodically be refined by adding participants from future studies using the same experimental paradigm.

## CRediT authorship contribution statement

**George C. O’Neill:** Formal analysis, Methodology, Writing - original draft. **Ayan Sengupta:** Formal analysis, Methodology, Writing - original draft. **Michael Asghar:** Formal analysis, Methodology, Writing - review & editing. **Eleanor L. Barratt:** Data curation, Formal analysis. **Julien Besle:** Methodology, Conceptualization, Writing - review & editing. **Denis Schluppeck:** Conceptualization, Formal analysis, Supervision, Writing - review & editing. **Susan T. Francis:** Conceptualization, Data curation, Methodology, Funding acquisition, Supervision, Writing - review & editing. **Rosa M. Sanchez Panchuelo:** Conceptualization, Data curation, Methodology, Funding acquisition, Supervision, Writing - review & editing.

## Figures and Tables

**Fig. A1:**
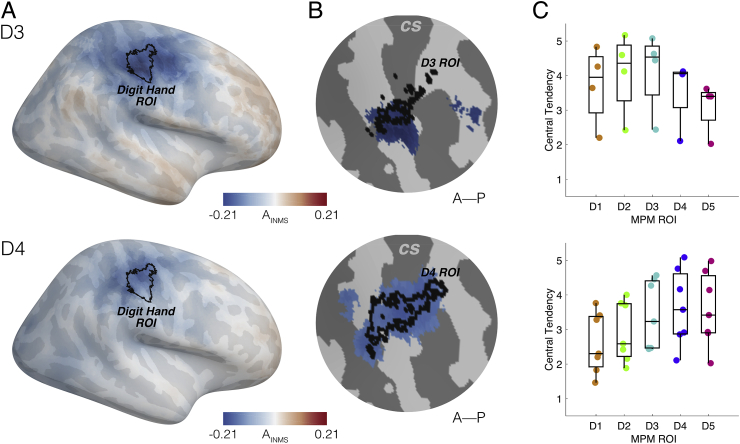
The application of the atlas to verify findings from a somatosensory study using MEG. A) Group-averaged functional activation images from experiments stimulating left D3 (top row) and left D4 (bottom row) showing the reduction of beta (13–30 ​Hz) oscillations during stimulation. The digit hand ROI is shown in black for reference. B) Zoomed in representations of the central sulcus, with an arbitrary threshold of 80% applied to the functional images and the corresponding digit ROIs from the MPM atlas in black. Note that the threshold is for illustrative purposes. C) The quantification of the central tendency scores within each of the atlas MPM ROIs for each experiment (unthreshold images). The experiment-median central tendency is correctly identified to be highest within the ROI corresponding to the digit stimulated in both subsets of experiments, implying the maximal reduction of beta power is specific to the digit simulated.

## Appendix Application of the probabilistic atlas to electrophysiological (MEG) responses to intraneural microstimulation

One of the purposes of the atlas is to assist in informing the sources of functional data in situations where precise knowledge of a subject’s digit areas is not available. For example, in electrophysiological imaging modalities such as magnetoencephalography (MEG) and electroencephalography (EEG), it is possible to localise digit areas using somatosensory evoked fields/potentials (Nakamura et al., 1998) but this often requires hundreds, if not thousands of repetitions of transcutaneous stimulation per digit using either electrodes (Meunier et al., 2001) or pneumatic stimulators (Jamali and Ross, 2013). This can be time consuming and in the case of the electrical nerve stimulation, often uncomfortable. If the atlas can be demonstrated to offer guidance on the somatotopic organisation of function from other experiments or be used as a spatial prior, then it has potential utility in functional studies.

Intraneural Microstimulation (INMS; Torebjörk and Ochoa, 1980; Vallbo, 1981) is the process of delivering a microampere-level stimulation to a single 1st order interneuron within an afferent nerve bundle, by inserting a laminated electrode subcutaneously and placing the exposed tip into a single axon. It allows to probe haptic touch at the quantal level; stimulation of the nerve gives a haptic sensation at the site of the mechanoreceptor and a functional response in the somatosensory cortex (Trulsson et al., 2001). It has been shown in a 7 T fMRI study where participants had both their median nerve stimulated using INMS and their digits mapped using a travelling wave paradigm, that the areas of maximal functional activation occurred within the expected digit area (Sanchez Panchuelo et al., 2016). We have also recently demonstrated that stimulation of a single 1^st^ order interneuron reliably induces a reduction in neural oscillatory power in S1 in the beta (13–30 ​Hz) band (O’Neill et al., 2019). However, in the latter study we did not have the probabilistic digit maps to corroborate that beta oscillations mapped to the correct digit representations in the cortex. In this exemplar study, we shall show that the probabilistic atlas suggests that beta oscillations which originate from receptive fields in separate phalanges of the hand are spatially separable and correspond directly to the cortical organisation of the digits seen in fMRI data.

### Methods

The datasets and experimental methods have been presented in (O’Neill et al., 2019), and so a summary outline of the data and processing methods follows. In this subset of the data, 3 participants volunteered to undergo INMS of the left median nerve using an in-house microneurography and microstimulation kit (Glover et al., 2017). When it had been confirmed that the spike discharge from a single mechanoreceptor in the left hand was detected by the electrode, a 1 ​s, 60 ​Hz spike train was delivered down the same electrode at 1 ​μA, with the current increased in 0.1 ​μA steps until a sensation was detected by the participant. If the sensation felt by the participant was confirmed to correspond a specific subclass of mechanoreceptor (as defined from the microneurography spike train characteristics), then concurrent microstimulation of the axon were administered whilst collecting neuromagnetic measurements from a 275-channel MEG system (CTF, Coquitlam, BC). Eighty microstimulation trials of (1 ​s, 60 ​Hz spike train, 10 ​s inter trial interval, up to 1 ​s jitter) were collected.

After quality control checks of the MEG data had been performed, the sensor-level data were filtered into the beta band (13–30 ​Hz) and source reconstructed onto 25,000 vertices of the subjects’ white matter surfaces, derived from their anatomical MR images using Freesurfer. Source reconstruction was performed using an LCMV Beamformer (Brookes et al., 2008), with dipole approximations calculated from a 3-shell BEM (Stenroos and Nummenmaa, 2016). From the source reconstructed data an ‘activation index’ image was generated by comparing oscillatory power during the stimulation period (0–1 ​s after stimulation onset) to a baseline period (8.9–9.9 ​s after stimulation onset). For each vertex **r**, the activation index *A*, was calculated as,

(A1) $$A\left(r\right)=\frac{P_{s}\left(r\right)-P_{b}\left(r\right)}{P_{s}\left(r\right)+P_{b}\left(r\right)},$$

where $P_{s}$ is the trial-averaged power during the stimulation window and $P_{b}$ is the trial-averaged power during the baseline period. Activation images were then warped to the MNI-305 cortical surface for subject averaging such that the MPM ROIs from the atlas could be overlaid to quantify the origin of the strongest change in oscillatory power, using the central tendency metric from Equation (3).

### Results

Microstimulation of 11 units corresponding to mechanoreceptors within the phalanges were successfully recorded in the MEG, 4 from digit 3 and 7 from digit 4. Figure A1 shows the group-average activation index images pooled into individual digits (D3 top row, D4 bottom row), with the digit hand ROI from the atlas overlaid in black. There is a reduction in beta oscillatory power (negative activity index) over the central sulcus which is consistent with other somatosensory studies (Cheyne, 2013; Hari and Salmelin, 1997; Hill et al., 2019). The reduction in power appears widespread but seems to overlap the digit hand ROI area, highlighting the precise, but diffuse nature of source-reconstructed MEG data. Applying an arbitrary threshold of 80% to the digit activation index images (Fig A1B) suggests that the locations of strongest reduction of power for D3 and D4 match the corresponding the digit MPM ROIs, following the lateral-medial (D1-D5) organisation of the digits. Figure A1C attempts to quantify this match by measuring the central tendency of each of the 11 (unthresholded) microstimulation response images, again pooled by mechanoreceptor origin (4 images for D3 and 7 for D4), to the MPM ROIs. While individual digit activation index images showed large variability in central tendency values (indicating that responses to the same digit stimulations were attributed to different MPM digit ROIs), the median central tendency across microstimulations of a given digit was maximal for the corresponding MPM digit ROI, showing that maximal reduction of power occurs in the expected atlas locations at the group level.
