# Supplementary material for: A probabilistic atlas of finger dominance in the primary somatosensory cortex
Source: Neuroimage. 2020 Aug 15;217:116880. doi: 10.1016/j.neuroimage.2020.116880 (PMC7339146; doi:10.1016/j.neuroimage.2020.116880)
Supplement: Multimedia component 1 [file mmc1.pdf]

A probabilistic atlas of finger dominance in the primary somatosensory cortex

Supplementary material

George C. O'Neill[ab†], Ayan Sengupta[acd†], Michael Asghar[a], Eleanor L. Barratt[a], Julien Besle[e], Denis Schluppeck[f], Susan T. Francis[ag*], Rosa M. Sanchez Panchuelo[a*]

[a]*Sir Peter Mansfield Imaging Centre, School of Physics and Astronomy, University of Nottingham, Nottingham, UK*
[b]*Wellcome Centre for Human Neuroimaging, Institute of Neurology, University College London, London, UK*
[c]*Wolfson Brain Imaging Centre, Department of Clinical Neurosciences, University of Cambridge, Cambridge, UK*
[d]*Department of Psychology, Royal Holloway, University of London, UK*
[e]*Department of Psychology, American University of Beirut, Beirut, Lebanon*
[f]*School of Psychology, University of Nottingham, Nottingham, UK*
[g]*NIHR Nottingham Biomedical Imaging Centre, University of Nottingham, Nottingham, UK*

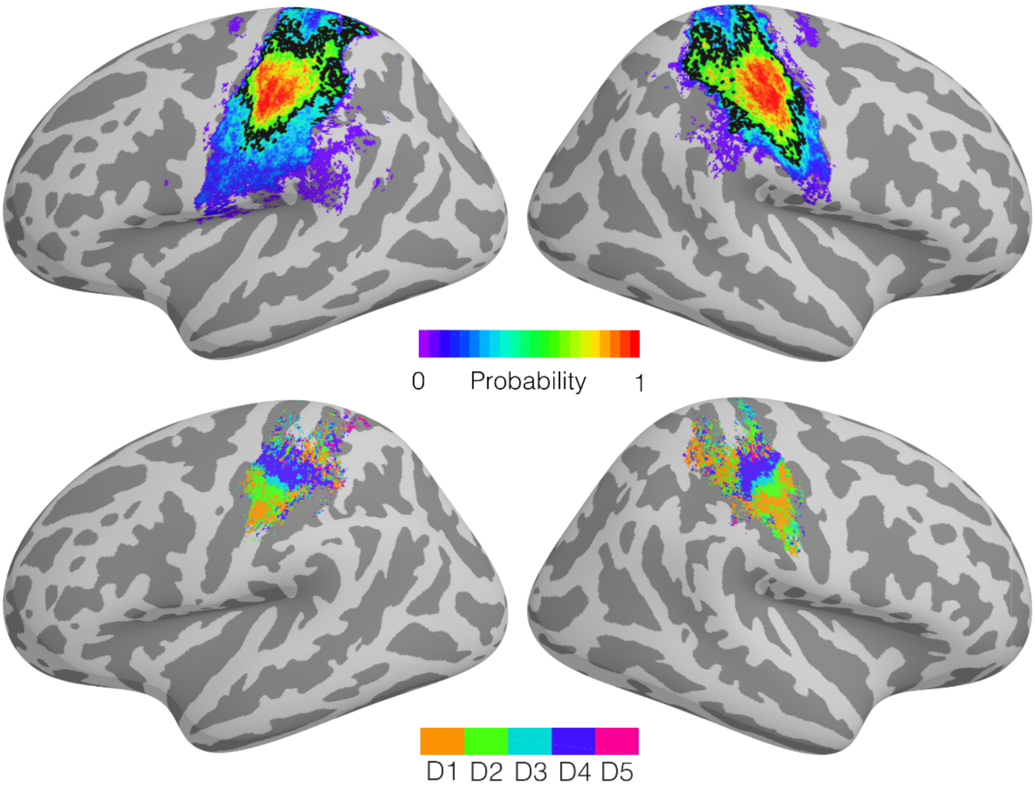

Figure S1: The results of the *automatic* masking method on the group level atlases. Top: The full probabilistic atlas (FPM) for all digits combined, the black boundary on each hemisphere represents the digit hand ROI, which is seen to extend more medially and laterally than the ROI derived from the *manual* masking. Bottom: The maximal probabilistic maps derived from the *automatic* masked data.

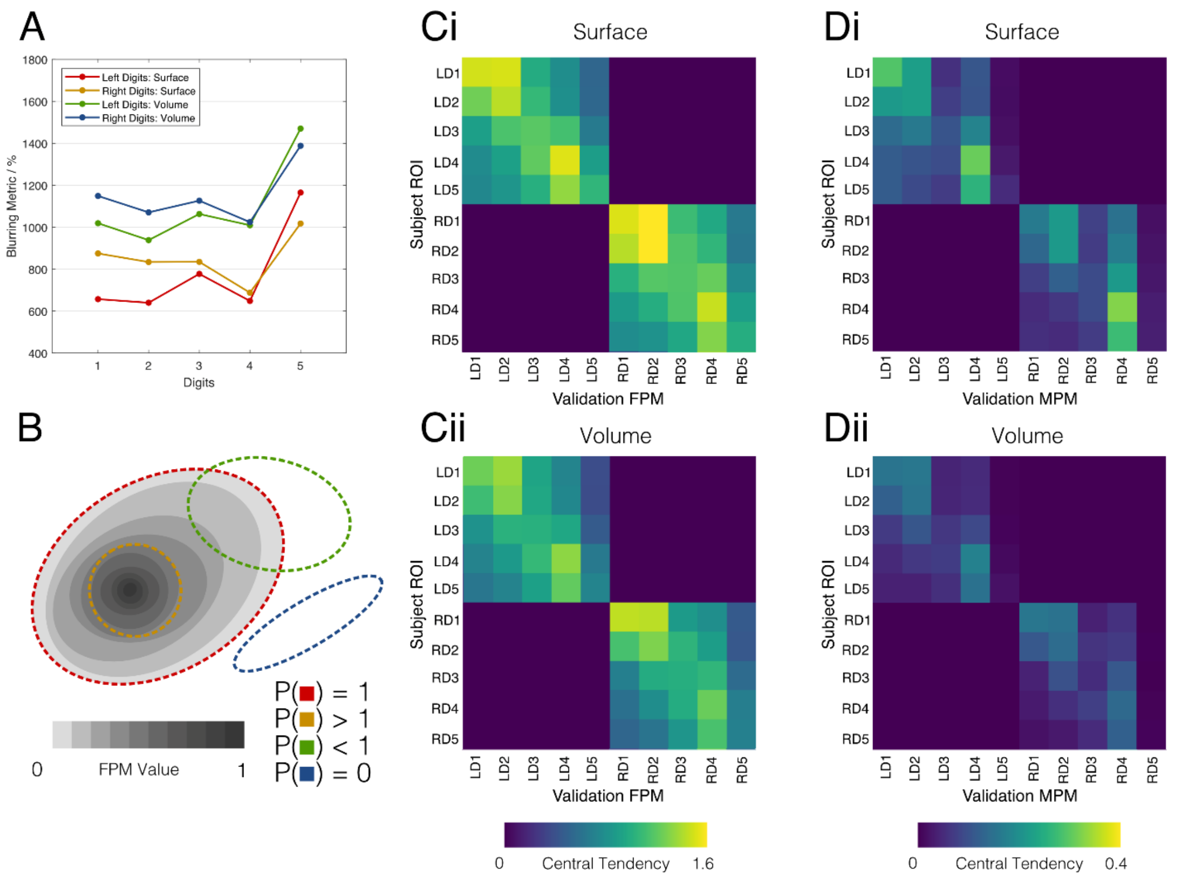

Figure S2: Blurring and leave-one-out results from the *automatic* masked data. A) The blurring metric for the full probability maps (FPMs) shows higher blurring results compared to the *manual* masked data. B) A cartoon diagram showing how the central tendency score, P, is affected by how a candidate ROI (dashed ellipses) overlaps an FPM. Here the scores are larger than one if an ROI is focalised over larger values of the MPM, a score of unity is achieved if it completely overlaps all non-zero values of the MPM, and it tends to zero the less it coincides. C) Central tendency scores from FPMs generated using the leave-one-out method, with the average scores from all 22 leave-one-out permutations shown. Maximal central tendency scores occur in the correct digits 5/10 times for the surface-based FPMs and 5/10 times for the volume based FPMs. For the MPMs the maximal central tendency scores occur for the correct digit 5/10 times for both surface and volume analysis.

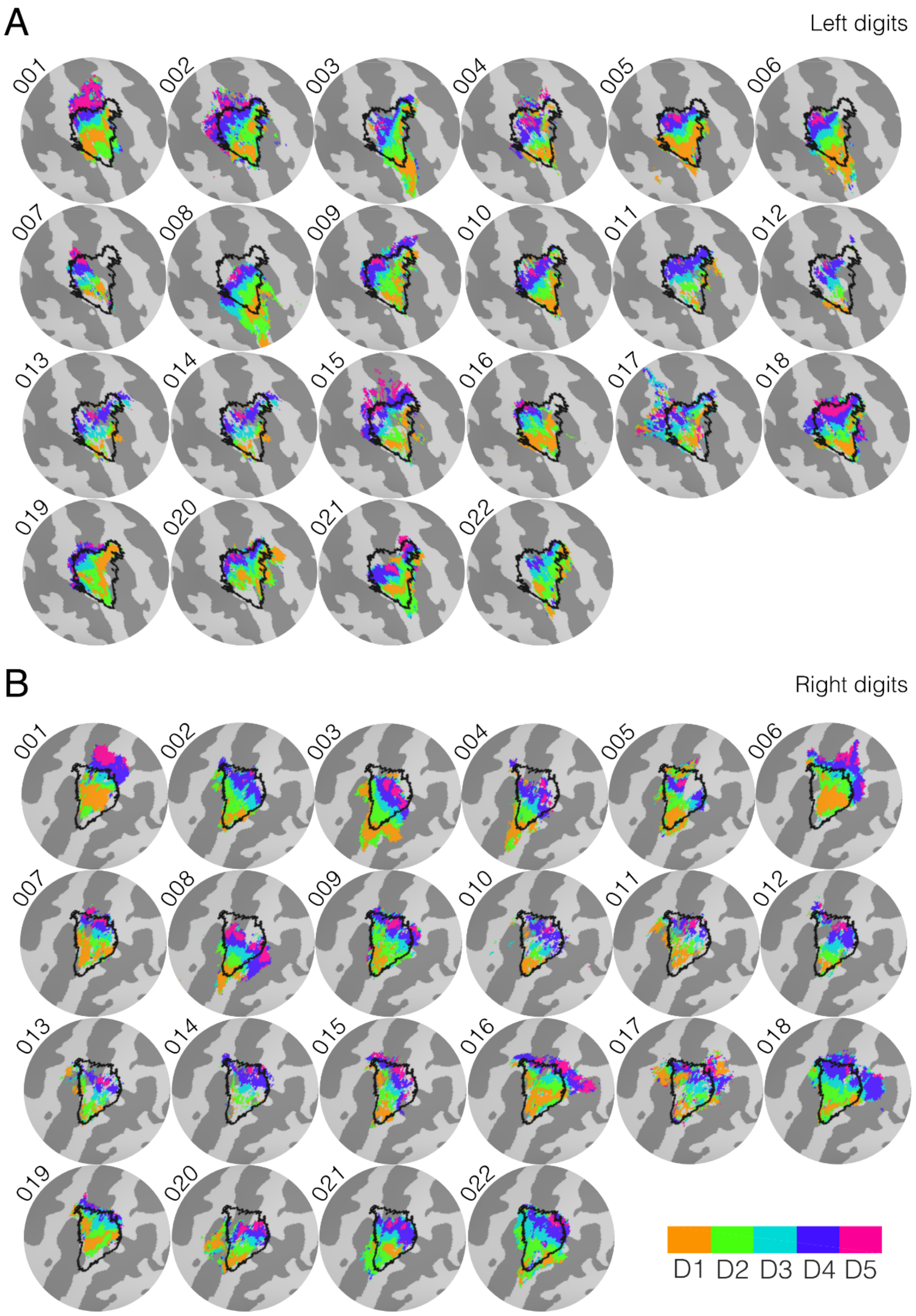

Figure S3: The individual digit maps of all 22 subjects, transformed into surface MNI space. Panel A shows the representation of the left digits in the right hemisphere and Panel B shows the right digits in the left hemisphere. In each plot, the group-level digit hand ROI is overlaid in black.

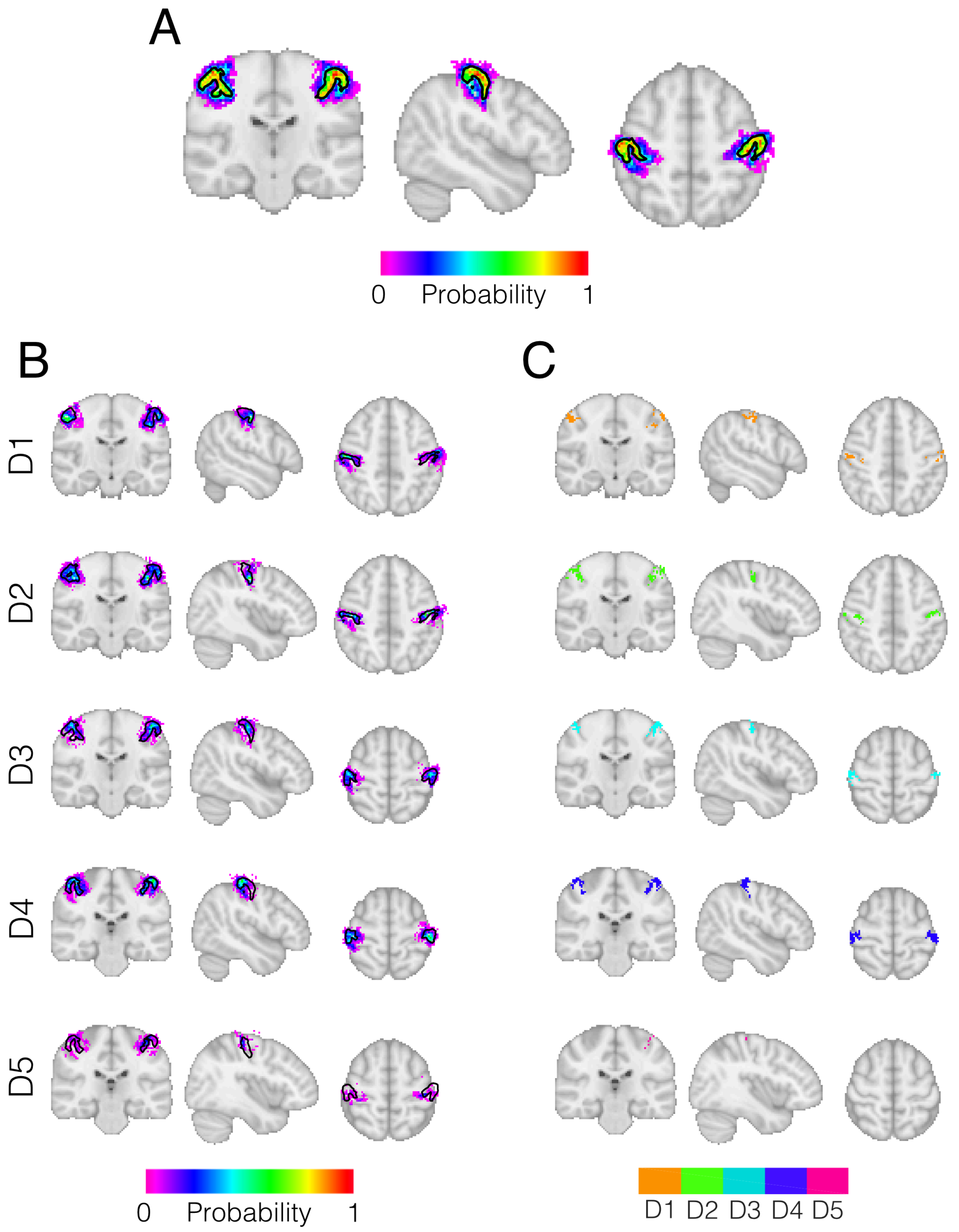

Figure S4: Visualisation of the volumetric atlases using *manual* masking. A) The combined FPMs of all digits, the black outline is the Digit area ROI as defined by the 50 % boundary. B) The FPMs of the individual digits. C) The MPMs of the individual digits.

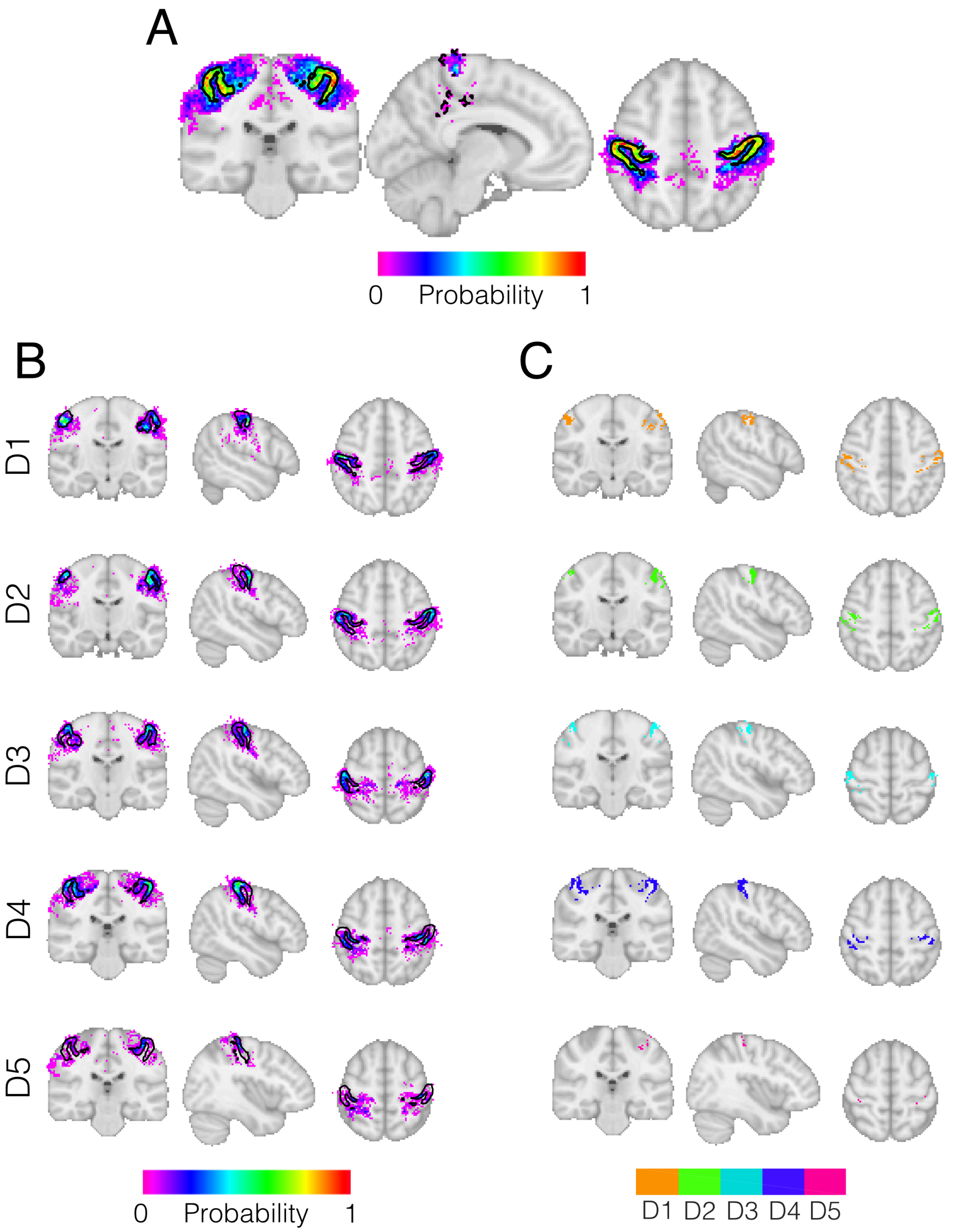

Figure S5: Visualisation of the volumetric atlases using *automatic* masking. A) The combined FPMs of all digits, the black outline is the Digit area ROI as defined by the 50 % boundary. B) The FPMs of the individual digits. C) The MPMs of the individual digits.

		Surface Atlas		Volumetric Atlas	
		Manual Mask	Automatic Mask	Manual Mask	Automatic Mask
Right Hand	D1	14	15	15	15
	D2	15	15	13	12
	D3	13	13	10	10
	D4	13	13	10	12
	D5	7	8	5	8
Left Hand	D1	15	16	12	12
	D2	16	17	15	15
	D3	13	12	12	12
	D4	15	16	13	14
	D5	9	10	7	8

Table S1: Maximal subject overlap values for each digit's FPM for each of the four processing pipelines.
